# Analysis of risk factors in thoracic trauma patients with a comparison of a modern trauma centre: a mono-centre study

**DOI:** 10.1186/s13017-020-00324-1

**Published:** 2020-07-31

**Authors:** Morris Beshay, Fritz Mertzlufft, Hans Werner Kottkamp, Marc Reymond, Ralph Alexander Schmid, Detlev Branscheid, Thomas Vordemvenne

**Affiliations:** 1 Department of General Thoracic Surgery, Protestant Hospital of Bethel Foundation, Burgsteig, 13 Bielefeld, Germany; 2 Department of Anesthesia and Intensive Care, Protestant Hospital of Bethel Foundation, Bielefeld, Germany; 3 Division of Accident & Emergency, Protestant Hospital of Bethel Foundation, Burgsteig, 13 Bielefeld, Germany; 4 Department of General Thoracic Surgery, University Hospital Berne, Berne, Switzerland

**Keywords:** Thoracic trauma, Lung injury, Injury Severity Score, Abbreviated Injury Scale, Lung Contusion, Rib fractures

## Abstract

**Abstract:**

**Objectives:**

Thoracic trauma (TT) is the third most common cause of death after abdominal injury and head trauma in polytrauma patients. Its management is still a very challenging task. The purpose of this study was to analyse the risk factors affecting the outcome in a high-volume trauma centre and the efficacy of a specialised trauma team in level 1 trauma centres.

**Patients and methods:**

Between January 2003 and December 2012, data of all patients admitted to the accident and emergency (A&E) department were prospectively collected at the German Trauma Registry (GTR) and thereafter retrospectively analysed.

Patients with chest trauma, an Injury Severity Score (ISS) ≥ 18 and an Abbreviated Injury Scale (AIS) > 2 in more than one body region were included. Patients were divided into two groups: group I included patients presenting with thoracic trauma between January 2003 and December 2007. The results of this group were compared with the results of another group (group II) in a later 5-year period (Jan. 2008–Dec. 2012). Univariate and multivariate analyses were performed, and differences with *p* < 0.05 were considered statistically significant.

**Results:**

There were 630 patients (56%) with thoracic trauma. A total of 540 patients (48%) had associated extrathoracic injuries. Group I consisted of 285 patients (197 male, mean age 46 years). Group II consisted of 345 patients (251 male, mean age 49 years). Overall 90-day mortality was 17% (*n* = 48) in group I vs. 9% (*n* = 31) in group II (*p* = 0.024). Complication rates were higher in group I (*p* = 0.019). Higher Injury Severity Scores (ISSs) and higher Abbreviated Injury Acale (AIS) scores in the thoracic region yielded a higher rate of mortality (*p* < 0.0001). Young patients (< 40 years) were frequently exposed to severe thoracic injury but showed lower mortality rates (*p* = 0.014). Patients with severe lung contusions (*n* = 94) (15%) had higher morbidity and mortality (*p* < 0.001). Twenty-three (8%) patients underwent emergency thoracotomy in group I vs. 14 patients (4%) in group II (*p* = 0.041). Organ replacement procedures were needed in 18% of patients in group I vs. 31% of patients in group II (*p* = 0.038).

**Conclusions:**

The presence of severe lung contusion, a higher ISS and AIS_thoracic_ score and advanced age are independent risk factors that are directly related to a higher mortality rate. Management of blunt chest trauma with corrective chest tube insertion, optimal pain control and chest physiotherapy results in good outcomes in the majority of patients. Optimal management with better survival rates is achievable in specialised centres with multidisciplinary teamwork and the presence of thoracic surgical experience.

## Introduction

Trauma continues to be a major public health problem worldwide, as it is associated with high morbidity and mortality in both developed and developing countries, with approximately 5.8 million deaths worldwide. Trauma has also been reported to be the leading cause of death, hospitalisation and long-term disabilities in the first four decades of life [1, 2]. Thoracic trauma comprises 20–25% of all traumas worldwide and constitutes the third most common cause of death after abdominal injury and head trauma in polytrauma patients [3, 4]. It directly accounts for approximately 25% of trauma-related mortality and is a contributing factor in another 25% of such cases [5]. Blunt thoracic injuries are more common than penetrating injuries, with the most frequent causes being motor vehicle accidents, falls and crush injuries [6].

Penetrating injury causes a laceration of anatomic structures in the trajectory of the weapon. A knife injury is typically limited to the length of the blade and the corresponding depth of the wound, assuming that the entire blade has penetrated in each instance [7]. Blunt injury, on the other hand, is much more common and usually compounded by dislocated skeletal fracture, which may lacerate the underlying viscera with sharp fragments. Although most injuries caused by blunt thoracic trauma are usually managed by chest tube drainage, surgical interventions are occasionally required in severe cases [8]. Blunt thoracic trauma, especially after motor vehicle accidents, is usually associated with a higher thoracic Abbreviated Injury Scale (AIS_thoracic_) score and Injury Severity Score (ISS). Therefore, individuals are susceptible to a higher risk of morbidity and mortality after thoracic trauma [9, 10].

Time management is a very important task, especially in patients with high ISS and AIS_thoracic_. During the first hour after hospital admission, thoracic vascular and neurologic trauma are the most common causes of death [5, 6]. The presence of an interdisciplinary team with high experience in anaesthesia, critical care and surgical disciplines, especially neurosurgery, trauma surgery, abdominal surgery and thoracic surgery, is mandatory to ensure high-quality management with low morbidity and mortality rates in these patients. The purpose of this study was to investigate the epidemiology, characteristics, incidence, management and risk factors affecting the outcome of polytrauma patients with chest injury admitted to our tertiary care facilities’ level I trauma centre in order to identify factors influencing management, possible complications and patient mortality.

## Patients and methods

### Study design

Data from all patients admitted to the A&E centre in our institution, Protestant Hospital of Bethel Foundation (EvKB), were collected prospectively using the German Trauma Register (GTR) database. Patients were either brought directly or transferred from another hospital. Once arrived, patients were taken to one of the available shock rooms, surveyed by the trauma teams according to the Manchester triage system (MTS), and thereafter managed according to the Advanced Trauma Life Support (ATLS) guidelines. The initial resuscitation initiated by the emergency transport team was continued or extended to intubation if necessary according to the stability of the vital signs. Those who were not intubated and had more stable vital signs underwent a complete medical history with detailed physical examinations. After initial chest and pelvic X-rays and stabilisation of the haemodynamic and respiratory situation, a CT scan was routinely performed (if there was no need for emergency operation at once) for further evaluation. After completion of the resuscitation and shock room procedures, patients were either admitted to the intensive care unit (ICU) for further stabilisation or underwent surgical interventions in the operating room (OR). Blood loss over chest tubes was recorded initially and continued over the following several hours. Prospective data collection was performed during the in-hospital time for age, gender, trauma mechanisms, type of transport, time of evacuation needed to free the patients, ISS, AIS, operative procedures, ICU procedures, length of intubation, complications, hospital stay and outcome, which were all recorded and retrospectively analysed. We analysed the data of all patients using our A&E collecting data system and data from the GTR. In this study, only data from the subgroup of patients with thoracic trauma over a 10-year period were analysed. To better compare and understand the differences, we decided to divide the patients into 2 groups in a 10-year period (the cut-off point was the implementation of a new division of thoracic surgery with two dedicated thoracic surgeons at our institution).

### Inclusion criteria

We included all patients with complete medical records who experienced thoracic trauma in a 10-year period. There were 630 patients included. Patients were divided into two groups; group I included patients presenting with thoracic trauma between January 2003 and December 2007 (no dedicated thoracic surgeons were available). The results of this group were compared with the results of another group (group II) in a later 5-year period (Jan. 2008–Dec. 2012, after the establishment of the division of general thoracic surgery). Forty-eight patients with thoracic trauma were excluded due to incomplete data.

### Statistical analysis

For univariable analysis, the Chi-squared (*X*^*2*^ test) or Fisher’s exact test and the numerical variables were compared by the *t* test or the Wilcoxon rank-sum test and used for categorical variables. Simple means were used for frequency and percentages for the categorical variables, while standard deviations (SDs) and the Mann-Whitney *U* test were used for the comparison of continuous variables. For multivariable analysis, a Cox regression model was used with a forward stepwise selection of covariates. Data analysis was performed using SPSS software (Version 16; SPSS, Inc., Chicago, IL, USA). Statistical differences with *P* < 0.05 were considered significant.

## Results

### Overview

Between January 2003 and December 2012, 1122 patients were admitted to our institution due to trauma. Of these, 1070 patients (95%) had blunt trauma. A total of 630 patients (56%) had thoracic trauma (TT). Group I (between Jan. 2003–Dec. 2007) consisted of 285, and group II (between Jan. 2003–Dec. 2007) consisted of 345 patients. Of these, 90 patients (14%) had isolated TT, but 540 patients (48%) had associated extrathoracic injuries. In total, 392 (34%) had two systems affected, and 311 patients (27%) had three or more organs affected. The associated injuries included 505 (80%) head and maxillofacial trauma cases, 271 (43%) extremity injuries, 127 (20%) abdominal injuries and 184 (29%) pelvic fractures; 67 patients (10%) had urological trauma, 45 (7%) had spinal injuries and 30 (3%) had considerable soft tissue injury (Table 1). Most of the patients in both groups had blunt thoracic trauma (88% vs. 92%). Fifty-five percent (*n* = 352) had loss of consciousness at the accident site with a Glasgow Coma Scale (GCS) ≤ 8 (57% in group I vs. 54% in group II). Most of the patients in both groups arrived intubated (84% in group I vs. 85% in group II). Eight percent had signs of aspiration in group I vs. 7% in group II. A gastric tube was inserted in 41% of group I patients vs. 46% of group II patients. A total of 68% (*n* = 196) of patients arrived with chest tubes in group I vs. 60% (*n* = 208) of group II. A new chest tube was inserted, the old one was corrected or a second chest tube was inserted in 10% (*n* = 29) of group I vs. 23% (*n* = 80) of group II. Young patients under 40 years of age were frequently exposed to severe thoracic injury with higher ISS and AIS_thoracic_ but showed lower mortality rates (*p* = 0.014). Overall morbidity was 52%, *n* = 331 (58% in group I vs. 43% in group II). In both groups, higher mortality rates were noticed in patients with higher AIS_thoracic,_ especially due to respiratory complications (*p* < 0.0001). In this subgroup of patients with higher AIS_thoracic_, a higher incidence of acute pulmonary failure that needed long-term respiratory support with or without extra-corporeal membrane oxygenation (ECMO) as well as extra-corporeal CO2 elimination using interventional lung assist (iLA) Novalung® (*p* = 0.031) was observed. ECMO and Novalung® were frequently used in group II (*p* < 0.001). The newly developed noninvasive ventilation (NIV) was frequently used in group II (*p* < 0.001). Early weaning from respiratory support was statistically higher in group II (*p* < 0.001).

**Table 1:** Type of associated non-thoracic injures

Accompanied injuries	Group I	Group II
Head and neck	231	254
Subdural hematoma (SDH)	167	178
Subarachnoid hematoma (SAH)	80	97
Brain laceration (BL)	16	21
Cerebral edema (CE)	198	222
Skull fracture (SF)	183	197
Maxillofacial fracture (MF)	48	61
Vertebral column fractures (VCF)	18	27
Abdomen	91	113
Splenic injury (SI)	41	46
Liver injury (LI)	26	31
Retro peritoneal haemorrhage (RPH)	15	23
Intestinal injury (II)	5	6
Urinary tract injury (UTI)	32	35
Skeletal system	278	322
Fracture of upper extremities (FUE)	36	42
Fracture lower extremities (FLE)	98	95
Pelvic fractures (PF)	86	98
Calvicula fracture (CF)	42	64
Scapula fracture (SF)	16	23
Soft tissue		
Open wound (OW)	104	113
Muscular laceration (ML)	18	24
Large subcutaneous hematoma (LSH)	29	35
Compartment syndrome (CS)	5	6
Peripheral nerve injury (PNE)	14	17
Vascular injury (VI)	12	16

Univariate and multivariate analyses showed higher mortality rates in patients with severe lung contusions (*p* < 0.001). In this subgroup, a higher occurrence of pneumonia and ARDS was noticed, especially in patients with more than 50% involvement of both lungs. The intubation time was 15 days in group I vs. 11 in group II. Eight percent (*n* = 23) of patients in group I underwent emergency thoracotomy vs 4% (*n* = 13) of patients in group II (*p* = 0.042). Many more VATS procedures as well as surgical chest wall fixations were performed in group II (*p* = 0.014) (Table 2). The mean ICU stay was 29 days (range 3–58) in group I vs. 34 days (range 2–67) in group II, and the mean hospital stay was 34 days (range 5–86) in group I vs. 31 days (range 6–94) in group II, with no significant difference. In addition, no significant difference was noticed between the two groups in terms of sex, type of transport, type of accident, blood transfusion or accompanying injuries of other organs. The overall 90-day mortality rate was 16% (*n* = 48) in group I vs. 9% (*n* = 31) in group II (*p* = 0.024).

**Table 2:** Thoracic operative procedures

Procedure	Group I	Group II
*Tracheostomy*	139	183
Dilatative	62	99
Surgical	77	84
*Chest tubes*	225	288
Single	122	136
Double	63	90
On both sides	40	62
Time of removal	18 days	12 days
*Emergency thoracotomy*	23	14
Open cardiac massage	7	2
Suture of lung laceration	4	5
Intercostal artery ligation	9	3
Repair of cardiac rupture	2	2
Repair of diaphragmatic rupture	1	2
*Elective thoracotomy*	15	2
Evacuation of hematoma	11	1
Decortication for empyema	4	1
Removal of foreign bodies	1	0
*VATS*	2	14
Evacuation of hematoma	1	9
Decortication for empyema	1	2
Removal of foreign bodies	0	3
*Thoracic wall stabilisation*	1	6

### ISS

The ISS is an anatomical scoring system that provides an overall score for patients with multiple injuries. Each injury is assigned an AIS and is allocated to one of six body regions (head, face, chest, abdomen, extremities (including the pelvis). Only the highest AIS in each body region is used. The 3 most severely injured body regions have their score squared and added together to produce the ISS. The ISS has values ranging from 0 to 75. If an injury is assigned an AIS of 6 (unsurvivable injury), the ISS is automatically assigned to 75. The ISS is virtually determined; moreover, it is the only anatomical scoring system in use and correlates linearly with mortality, morbidity, hospital stay and other measures of severity. Its weaknesses are that any error in AIS scoring increases the ISS error, many different injury patterns can yield the same ISS, and injuries to different body regions are not weighted. Additionally, as a full description of patient injuries is not known prior to full investigation and operation, the ISS (along with other anatomical scoring systems) is not useful as a triage tool. A total of 555 patients (88%) with severe thoracic trauma had ISS ≥ 16. The ISS was summarised and compared between both groups (Table 3). The mean ISS was 32 (28 in group I vs. 33 in group II). Univariate and multivariate analyses showed higher morbidity with a higher risk of respiratory and cerebral complications in patients with ISS > 30 (*p* = 0.026). A higher mortality rate in patients with ISS > 30 (*p* < 0.0001) was also identified.

**Table 3:** Injury Severity Score (*ISS)*

ISS	Group I	Group II
< 16	33 Died: 0 Survived: 33	42 Died: 0 Survived: 42
16–30	188 Died: 11 Survived: 177	226 Died: 8 Survived: 218
> 30	64 Died: 36 Survived: 25	77 Died: 20 Survived: 57

### AIS_thoracic_

**Fig. 1:**
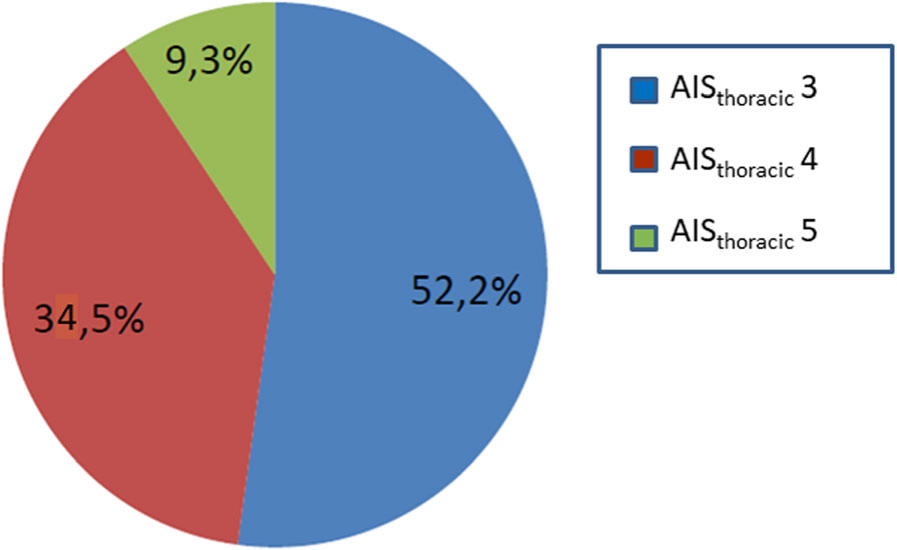
Distribution of AIS_thoracic_

The AIS_thoracic_ is an anatomical-based coding system created by the Association for the Advancement of Automotive Medicine to classify and describe the severity of a specific individual injuries. There were 124 patients (43%) with AIS_thoracic_ scores of 4 and 5 in group I compared to 154 (45%) in group II (Fig. 1). A summary of the patient’s AIS_thoracic_ score in both groups was analysed and compared (Table 4). A higher mortality rate in patients with AIS_thoracic_ > 3 (*p* < 0.0001) was identified in both groups.

**Table 4:** AIS_thoracic_ and outcome

AIS	Description	Chest	Head	Abdomen	Extremities	Group I	Group II	*p* value
0	Not injured	0	125	503	175	0	0	
1	Minor	30	178	47	135	14 Died: 0 Survived: 14	18 Died: 0 Survived: 18	P > 0.5
2	Moderate	87	146	20	89	59 Died: 1 Survived: 58	68 Died: 0 Survived: 68	P < 0.05
3	Serious	169	118	38	217	94 Died: 7 Survived: 90	108 Died: 2 Survived: 106	P < 0.05
4	Severe	278	14	16	10	107 Died: 28 Survived: 79	125 Died: 12 Survived: 113	p > 0.05
5	Critical	66	5	6	4	17 Died: 12 Survived: 4	26 Died: 17 Survived: 9	P < 0.05

### Mechanism of injury

The most frequent mechanism of injury in the overall study population was road traffic accidents (RTAs), at 57%. Car crashes were the most frequent cause among RTAs (36.4%), followed by motorcycle crashes (16.8%) and injured pedestrians (7.4%). Falls made up for most of the remaining injuries (23.9%). Injuries caused by bicycles were represented by 7.3%. There were 6% (*n* = 37) of cases in which no defined mechanism could be obtained.

### Type of associated non-thoracic injuries

Associated extrathoracic injuries were most frequent in the head and neck region, the lower extremities and then upper extremities, followed by abdominal injuries and then pelvic injuries. An overview is summarised and compared in both groups (Table 1).

### Type of thoracic injuries

The most common thoracic injuries were lung contusions followed by haemothorax, rib fractures and then pneumothorax. The prevalence of common thoracic injuries was analysed and compared in both groups (Table 5).

**Table 5:** Type of thoracic injuries

Type of thoracic injury	Group I	Group II
Blunt trauma	217	295
Penetrating trauma	12	9
Soft tissue injuries in total	62	119
Thoracic wall laceration	7	10
Thoracic wall hematoma/contusion	15	23
Diaphragmatic injury	6	17
Subcutaneous emphysema	34	69
Skeletal injuries total	227	291
Rib fracture:		
Single	12	16
Multiple	157	186
Flial chest	19	27
1st rib fracture	5	9
Sternal fracture	6	11
Vertebral fracture	28	42
Thoracic trauma total	285	345
Lung contusion	209	296
Hemothorax	198	286
Pneumothotax	145	221
Tension pneumothorax	14	17
Intra pul. hematoma	23	34
Lung laceration	22	41
Intra pul. pneumocyst	7	16
Cardiac/vascular injuries total	30	68
Myocardial contusion	18	41
Pericardial effusion	5	8
Pericardial tamponade	2	5
Myocardial perforation	1	2
Aortic rupture	3	5
Pulmonary artery injury	0	3
Lung veins injury	1	2
Azygus vein injury	0	2
Mediastinal injuries total	51	86
Tracheal/broncheal rupture	2	6
Oesophageal rupture	2	2
Mediastinal hematoma	15	33
Pneumomediastinum	27	41
Thoracic duct	2	0
Phrenic nerve	3	4

### Degree of lung contusion

A total of 322 patients (51%) had mild lung contusions, 138 patients (22%) had moderate lung contusions, and 94 patients (15%) had severe lung contusions. The presence of pneumatoceles and other signs of lung lacerations were frequently seen in both moderate and severe lung contusions but did not show significant differences in complication or mortality rates.

**Fig. 2:**
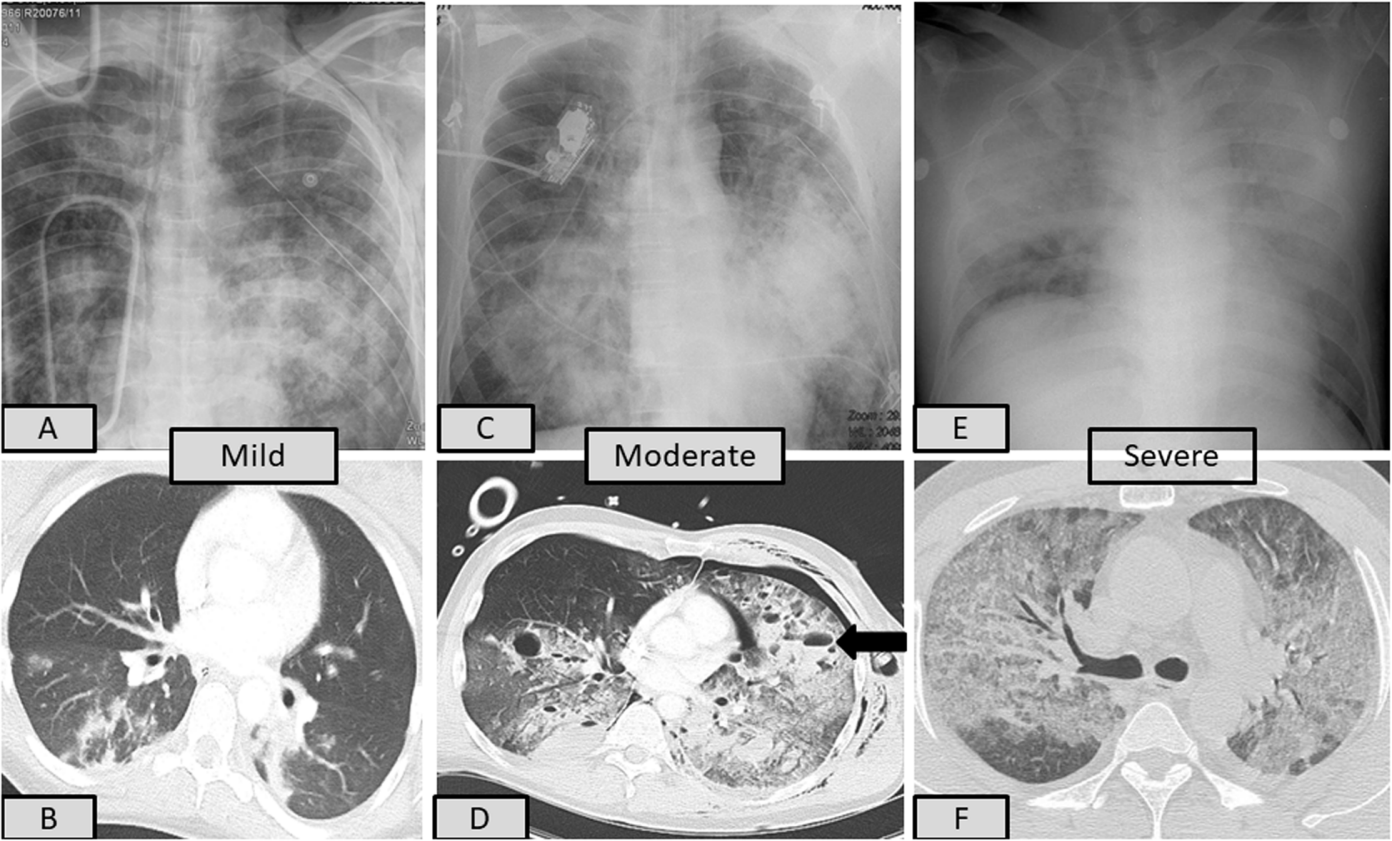
Distribution of pulmonary contusion on X-ray and the corresponding CT-scan. **a** Chest X-ray showing mild contusion. **b** CT scan showing mild contusion (mainly on the right side). **c** Chest X-ray showing moderate contusion (mainly on the left side). **d** CT scan showing moderate contusion with the presence of multiple pneumatoceles (arrow). **e** Chest X-ray showing severe contusions on both sides. **f** CT scan showing severe contusion on both sides

The degree of lung contusions was classified according to the findings on CT scans of the lung. The lung contusion volume was calculated according to the relation of the affected lung volume to the non-affected lung volume (Fig. 2). The following classification was performed:

- Mild lung contusion: less than 20% of the whole lung volume was affected. There were 146 such patients in group I (two patients died, 1.3%) and 176 in group II (one died, 0.56%).
- Moderate lung contusion: 20–50% of the whole lung volume was affected. There were 65 such patients in group I (four died, 6%) and 73 in group II (five died, 6.8%)
- Severe lung contusion: more than 50% of the whole lung volume was affected. There were 33 such patients in group I (18 died, 54%) and 61 in group II (13 died, 21%); (*p* < 0.001) (Table 6).

**Table 6:** Effects of pulmonary contusion on the outcome

PC	Mild PC	Moderate PC	Severe PC	*p* value
*N* (group I vs. group II)	322 (146 vs. 176)	138 (65 vs. 73)	94 (33 vs. 61)	
Pneumonia	2	6	14	*p* > 0.05
Intubation time	1 (1–4 vs. 1–2)	8 (3–11 vs. 2–10)	21 (8–44 vs. 6–46)	***p*< 0.05**
ARDS	0	2 (2 vs. 0)	33 (26 vs. 7)	***p*< 0.05**
SIRS	0	1	5	*p* > 0.05
Sepsis	1	3	9	*p* > 0.05
MODS	0	0	4	*p* > 0.05
ICU time (days)	3 (1–5 vs. 1–3)	11 (4–17 vs. 2–15)	32 (19–61 vs. 14–51)	***p*< 0.05**
ECMO (days)	0	0	4 (1 vs. 3)	*p* > 0.05
Jet ventilation	0	0	18 (5 vs.13)	***p*< 0.05**
Thoracic surgery procedures	0	3 (1 vs. 2)	9 (2 vs.3)	*p* > 0.05
Mortality	1 (1 vs. 0)	7 (5 vs. 2)	19 (14 vs. 5)	***p*< 0.05**

### Morbidity/mortality

Complications were documented, analysed and compared in both groups. Twenty-two patients (7%) had nosocomial pneumonia in group I vs. 10 patients (2.8%) in group II (*p* = 0.033). More patients with atelectasis in group I (*n* = 34) than in group II (*n* = 12) were observed (*p* = 0.019). ARDS was more common in group I (*n* = 16) than in group II (*n* = 5) (*p* = 0.016). Organ replacement procedures, e.g. ECMO, NovaLung®, Jet ventilation and renal dialysis, were frequently used in group II (*n* = 109) compared with group I (*n* = 51) (*p* = 0.038). The overall 90-day mortality was 13% (*n* = 79). Higher mortality in group I (*n* = 48) than in group II (*n* = 31) with a lower incidence in younger patients under 40 years old (*p* = 0.024 and *p* = 0.014, respectively) was noticed. Other complications, such as reoperation, pleural empyema, cardiovascular events, lung emboli and neurological complications, were higher in group I but did not show significant differences (Table 7).

**Table 7:** Morbidity and mortality in both groups

Outcome	Group I	Group II	*p* value
*N*	285	345	
Pneumonia	66	24	***p*< 0.05**
Atelectasis	51	13	***p*< 0.05**
Wound infection	9	6	*p* > 0.05
ARDS	51	44	*p* > 0.05
SIRS	72	66	*p* > 0.05
Sepsis	44	23	***p*< 0.05**
MODS	24	12	*p* > 0.05
Intubation time (days)	18	11	***p*< 0.05**
ICU time (days)	28	31	*p* > 0.05
Mortality	48	31	***p*< 0.05**

## Discussion

Thoracic trauma is one of the leading causes of death in Germany and many other countries worldwide. It is responsible for one-third of all traumatic deaths in the USA. Blunt thoracic trauma is much more common than penetrating trauma and is increasing worldwide (1, 13). We agree with Veysi et al. that patients with higher ISS and AIS_thoracic_ show a significant risk of developing multi-organ failure (MOF), with higher morbidity and mortality rates. In contrast to the findings of Chrysou et al., our results showed that the severity of chest trauma, based on the AIS with the presence of severe lung contusion, correlates with the hospital and ICU lengths of stay, the time of mechanical ventilation, complications and mortality rates. We explain this finding by the fact that patients with an AIS_thoracic_ score of 4 and especially 5, even without other associated injuries such as head and abdominal injuries, suffered serious complications, especially MOF. The use of ECMO, NovaLung® and Jet ventilation are effective tools to overcome temporary acute cardiac and respiratory failures [11, 12]. Our results showed the efficacy of using new ventilator strategies with early weaning as well as the generous use of NIV equipment on morbidity and mortality. The causes of death were ARDS, sepsis, aspiration and multiple organ failure. Our results are in agreement with other reports that younger patients (< 40 years old) have lower mortality rates, although there was no significant difference in ISS, thoracic AIS or severity of lung contusion in different age groups [13]. The overall mortality in group II was significantly lower than that in group I and previously reported studies [12, 14]. We explain this finding by the fact that there were frequent uses of EMO, NovaLung®, Jet-Ventilators and NIV in group II. Over the last decade, there has been a huge change in the strategies of ventilation and the application of NIV or other devices in the field of trauma management. Furthermore, there were more sophisticated thoracic surgical procedures performed in group II, such as early fixation of rib fractures and the use of minimally invasive procedures such as VATS or mini-thoracotomies instead of standard thoracotomies. Therefore, the better outcome in group II could be explained by establishing a well-developed network of prehospital trauma management, establishing an independent A&E unit, improving intensive care resuscitation, using sophisticated devices to replace organ failure and establishing an independent department of thoracic surgery. Altogether, these findings represent a new synergetic system that may enhance the level of trauma care and could result in better survival for trauma patients. Interestingly, approximately one third of all deaths in our study were attributed to chest trauma itself, showing the importance of immediate thoracic surgical treatment if possible to reduce unnecessary emergency thoracotomies in polytrauma patients. However, it must also be considered that a significant proportion of deaths attributed to severe chest trauma occur in the prehospital setting [15]. Although the majority of our patients with blunt chest injury could be treated without surgery (85.8%), most of them required chest tube thoracostomy (93%). In our study, 64% of the chest tubes were placed through the emergency team at the injury site or during transport due to unstable haemodynamic conditions, especially in cases of tension pneumothorax, suspicion of pneumothorax or haemothorax. In accordance with other studies, only 8% of our polytrauma patients required surgical treatment for their chest injuries due to unsuccessful non-operative treatment, such as non-resolving pneumothorax despite thoracic drainage, late haemothorax, persisting air leaks or late pleura empyema. Patients with bilateral flail chest with paradoxical movement and/or the need for positive pressure ventilation for more than 48 h were considered as an indication for immediate surgery [10, 16]. In the case of flail chest, early stabilisation is an effective way to avoid long-term intubation [17].

**Fig. 3:**
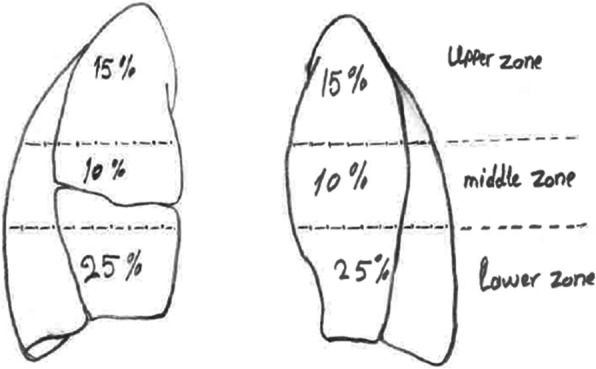
Schematic illustration of pulmonary contusion

The degree of lung contusion plays an important role in developing respiratory complications, such as pneumonia and acute respiratory distress syndrome (ARDS). Our results support the hypothesis by Clark et al. that the presence of severe pulmonary contusion is one of the important prognostic factors leading to long-term intubation with a risk of developing pneumonia and ARDS. In their series, the mortality rate was more than doubled when a combined pulmonary contusion and flail chest were present [18]. Accordingly, we may assume that a new classification for lung contusion using the affected zone is needed (Fig. 3).

We identified a high incidence of left-sided ruptured diaphragms, similar to that in other publications [17, 19, 20]. In contrast to the findings of Rodriguez and colleagues, there were fewer patients who had right-sided diaphragmatic rupture in our study. Small diaphragmatic rupture is usually difficult to diagnose, and many cases remain occult, especially on the right side [21].

Emergency thoracotomy (ET) plays an important role in penetrating trauma but continues to be controversial in blunt trauma. All patients (*n* = 4) in this study who had absent vital signs in the emergency room died despite aggressive resuscitative measures, including emergency room left thoracotomy. ET might be effective in the treatment of a ruptured cardiac chamber or severe pulmonary parenchymal laceration [22]. Our general philosophy in case of doubt is to perform ET as it is better to err on the side of resuscitation rather than declaring the patient dead on arrival. However, we agree with other reports that if the patient has no vital signs at the scene or has been without vital signs for 30 min, this represents a non-salvageable patient [22, 23]. Emergency thoracotomy was frequently performed in group I compared with group II, and these ratios are similar to previously published results [3, 11, 24]. Our results showed a significant decrease in the number of ETs in group II in the presence of a dedicated thoracic surgeon. This demonstrates the importance of specialised thoracic surgeons at high-frequency trauma centres.

VATS as a minimally invasive surgery is an effective method to explore intrathoracic injuries in stable patients. Many reports have identified the efficacy of VATS in cases of thoracic trauma. We agree with Freeman et al. regarding the indications for VATS in cases of an abnormal chest radiograph, associated intra-abdominal injuries, a high-velocity mechanism of injury, an entrance wound inferior to the nipple line or scapula, and a right-sided entrance wound [17, 25, 26].

Atelectasis and pneumonia are two of the most common causes of death in ICU patients with multiple injuries, and every effort has to be made to manage these conditions. The development of nosocomial pneumonia, especially in patients with known COPD or emphysema, has an adverse prognostic effect on the outcome. At our institution, the concept of “hit hard and early” using bronchoscopic examination and broad-spectrum antibiotics has shown efficacy. In this study, there was a significantly lower mortality rate (4%) due to pneumonia in group II than in other series [27]. We postulate that early and complete drainage of haemothorax or pneumothorax, repeated bronchoscopy, early mobilisation, aggressive analgesia, vigorous physical and respiratory therapy, and early use of antibiotic therapy in case of infection are the most important factors to improve the outcome of blunt thoracic trauma.

## Conclusions

 Mortality rates in polytrauma patients with blunt chest trauma correlate with the severity of chest injury. High ISS (greater than 30), high AIS_thoracic_ score (greater than 4), advanced age and severe lung contusion were independent predictive factors for mortality in our study. Surgeons with thoracic surgery experience play an important role in the trauma team. Management of blunt chest trauma with corrective chest tube insertion, optimal pain control and chest physiotherapy resulted in good outcomes in the majority of patients.

### Study limitations

Although the data of all trauma patients were prospectively collected through the German trauma register, this study was dependent on a retrospective analysis. This investigation represents a mono-centre experience with a newly evolving ICU, A&E unit and thoracic surgery department. To validate the results shown herein, multicentric prospective studies are needed.

## Data Availability

All data generated or analysed during this study are included in this published article.

## Appendix

**Conference discussion**

***Marcelo Jimenez (Salamanca, Spain)***

Congratulations on your nice presentation. Thoracic trauma is a frequent cause of admission in our daily practice, and you have presented a great new series of patients. I understand that you separated both groups because you had started a new protocol with thoracic surgeons in your hospital and to demonstrate the performance of the thoracic surgical team managing this kind of intervention. I have two questions for you. You had mentioned that some patients had isolated trauma and that some other patients had polytrauma. Have you considered comparing the impact of the morbi-mortality of those patients with polytrauma? Because sometimes the cause of death is not thoracic trauma (it is head trauma or whatever), have you considered comparing both?

***Dr. Beshay****:*

Thank you very much for this good question. We performed a detailed analysis of these patients with thoracic trauma and those with polytrauma. In this study, we included only patients in whom the leading trauma or leading problem was chest trauma. Many of them had the head and neck or other systemic injuries, but those were not a leading cause. Therefore, most of the patients recovered from other injuries, such as brain trauma or extremities, very well, and we found that the mortality among these patients who had thoracic trauma as a leading trauma was due to thoracic trauma and not due to brain damage or other injuries. Other patients who had, for example, a mild thoracic injury were not analysed because they went to the other departments.

***Dr. Jimenez:***

My last question is whether in your analysis you take into account the Injury Severity Score and the Abbreviated Injury Score, but, as you know, these scales underestimate the mortality depending on open or closed trauma. Did you take this item into account when you analysed both groups?

***Dr. Beshay:***

There were very few patients with penetrating trauma in our study. Therefore, we did not look at open chest trauma separately because there was a very small number of patients. There were approximately 5% in both groups with open chest trauma; therefore, it is difficult to determine any significant difference.

***Dr. Jimenez:*** Thank you.

## Abbreviations

A&E: Accident and emergency
AIS: Abbreviated Injury Scale
ARDS: Acute respiratory distress syndrome
CT: Computed tomography
ET: Emergency thoracotomy
ICU: Intensive care unit
ISS: Injury Severity Score
GCS: Glasgow Coma Scale
GTR: German Trauma Register
MTS: Manchester Triage System
OR: Operating room
RTAs: Road traffic accidents
TT: Thoracic trauma
